# Is arteriovenous fistula a risk factor for squamous cell carcinoma? Evaluation at a University Hospital^☆^

**DOI:** 10.1016/j.abd.2023.07.015

**Published:** 2024-05-31

**Authors:** Ariany Tomaz de Aquino Saran Denofre, Thais Helena Buffo, Rafael Fantelli Stelini, Maria Leticia Cintra, Renata Ferreira Magalhães

**Affiliations:** a Discipline of Dermatology, Medical Sciences College, Universidade Estadual de Campinas, Campinas, SP, Brazil; b Department of Pathology, Medical Sciences College, Universidade Estadual de Campinas, Campinas, SP, Brazil

Dear Editor,

Squamous cell carcinoma (SCC) is the most common neoplasm in transplant (TX) patients, when it is more aggressive and presents a worse prognosis.^1^, ^2^ In kidney transplant recipients the occurrence of SCCs is over or close to arteriovenous fistulas (AVF), whether they are active or not. Two mechanisms are proposed: impaired immune response due to overload of the lymphatic system of the affected extremity and, facilitation of HPV-related tumors secondary to surgical trauma due to fistula formation, repetitive punctures, and excision of multiple tumors.^3^ Moreover, 3.7%‒5% of dialysis patients develop limb ischemia, leading to oxidative stress that can potentiate carcinogenic factors for the development of SCC.^3^ After kidney transplantation, many patients remain with an AVF and start using immunosuppressants. These medications add a risk of up to 100× for the development of SCC.^4^, ^5^, ^6^ There is also a greater susceptibility to the HPV virus, with DNA from the virus being found in 80% of SCCs in immunosuppressed patients.^6^

Data from the literature show a high incidence of skin tumors in kidney transplant recipients, but there are no studies that analyze whether the fistula influences tumor development. Therefore, this project aims to report three cases of SCCs appearing close to fistulas, explaining their challenges and analyzing the occurrence of these tumors, whether this association is true or not.

## Materials and methods

Three cases operated at Unicamp Dermatology Service from 2020 to 2022 were selected. A total of 118 patients who had undergone follow-up at the Nephrology and Dermatology Units were also selected to collect data from their medical records, and were divided into groups based on their history of fistula: never had one, inactive or active. In the case of patients with a history of fistula (inactive or active), this upper limb was chosen for analysis, and the upper limb of those who had never had a fistula was chosen at random. A *post-hoc* test was performed to investigate multiple comparisons between fistula history status in the groups. Afterwards, it was decided to group patients with active and inactive fistulae and compare them with patients who had never had a fistula using the Mann-Whitney test. All analyses were performed using R software.

## Results

### Case reports

**Figure 1:**
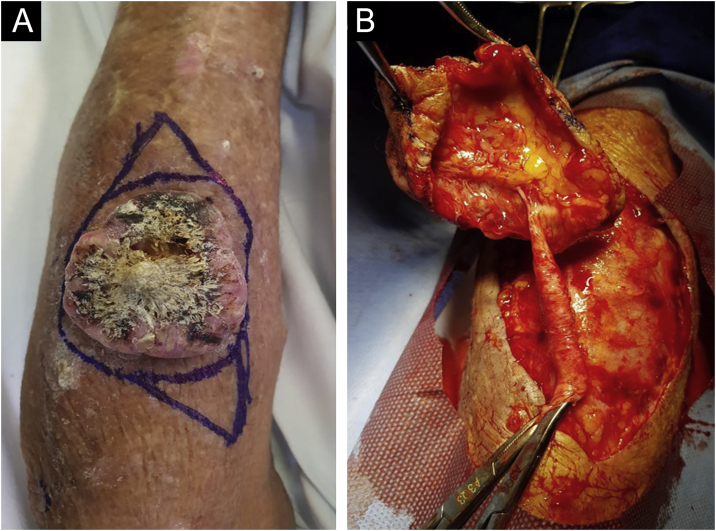
(A) Clinical appearance of the tumor. (B) Intraoperative image showing large vessel entering the excised lesion.

**Figure 2:**
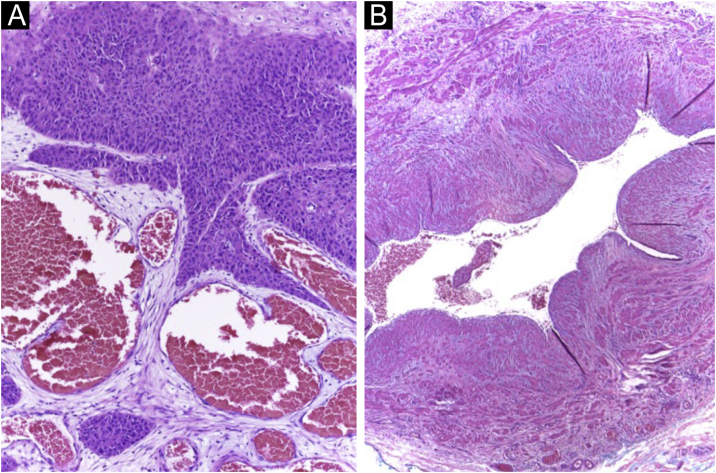
Histopathology of the tumor in Fig. 1 (Hematoxylin & eosin staining). (A) Neoplasm in close contact with vessels derived from the fistula. (B) Disconnected arteriovenous fistula removed surgically.

A 68-year-old male patient, kidney TX recipient (2011), with a 5.0 x 4.0 cm tumor on the left forearm over an inactive AVF. Intraoperatively, an inactive fistula vessel was observed in close contact with the tumor (Figure 1, Figure 2).

**Figure 3:**
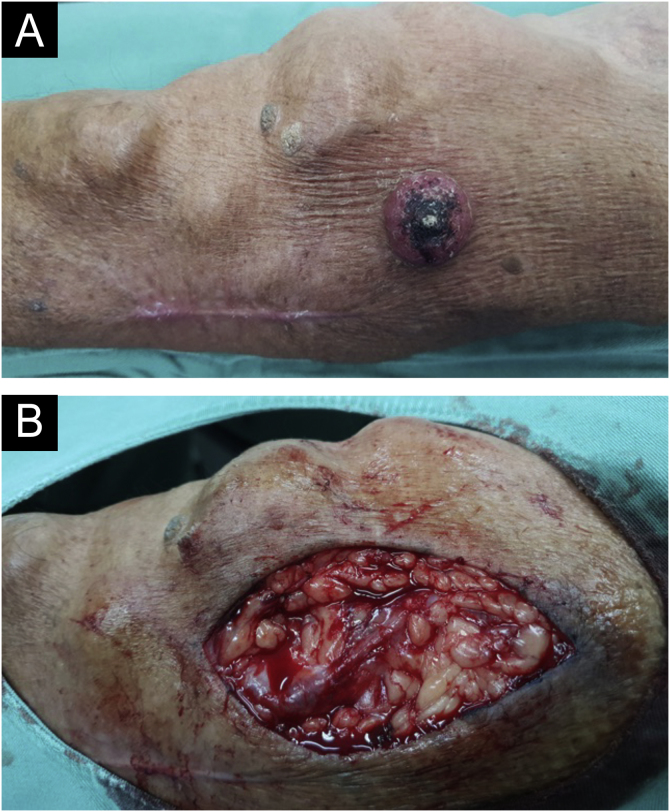
(A) Tumor close to an AVF. Note the scar from a previous excision of SCC. (B) Intraoperative image revealing a medium-sized vessel close to the operated territory.

A 69-year-old male patient, kidney TX recipient (2009), with a 3.5 × 3.0 cm tumor on the left forearm close to an active AVF (Fig. 3).

**Figure 4:**
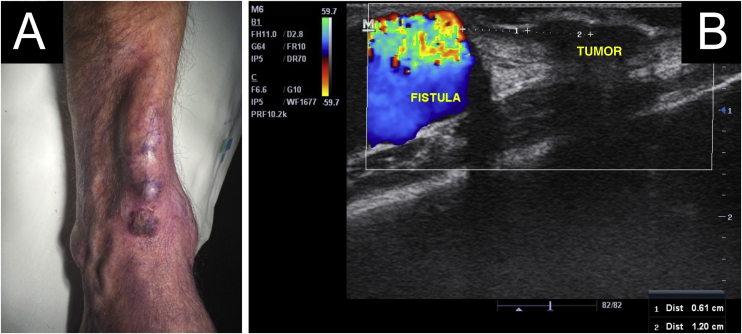
(A) Tumor located distally to an active AVF. (B) Dermatological ultrasound (Mode B and Mindray Doppler, 16 MHz linear probe). Tumor 0.6 cm away from the active fistula.

**Figure 5:**
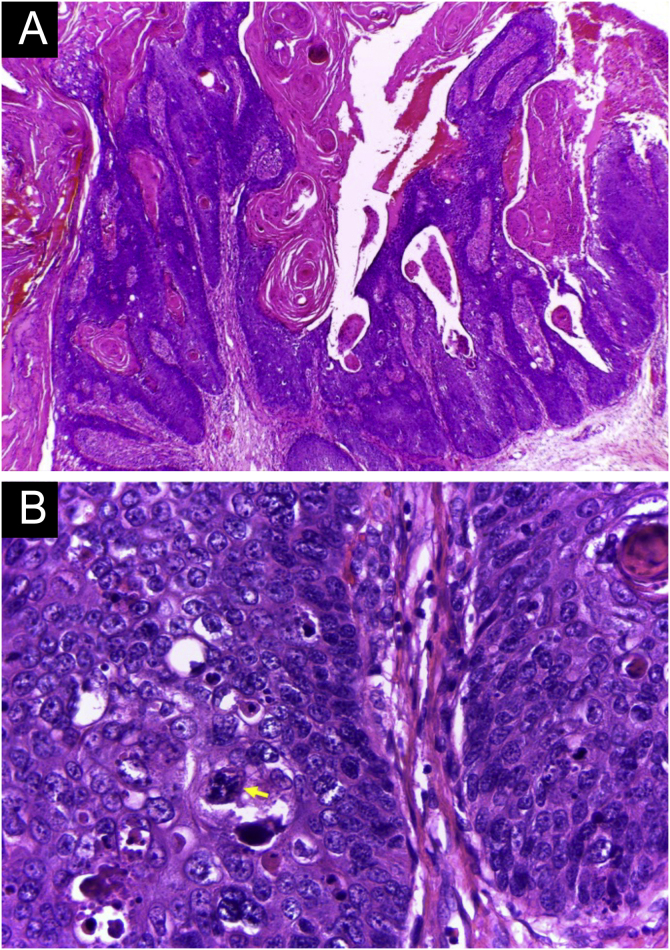
Histopathology of the tumor seen in Fig. 4 (Hematoxylin & eosin staining). (A) Exophytic neoplasm, with papillomatosis and abundant hyperkeratosis, giving it a verrucous appearance (probably due to HPV, secondary to immunosuppression). (B) Atypical hyperchromatic nuclei and an atypical mitoses (yellow arrow).

A 64-year-old kidney TX recipient (2017), with a 1.3 × 1.0 cm tumor on the right forearm, distal to an active AVF (Figure 4, Figure 5). High-frequency Doppler Ultrasound was performed (Fig. 4) demonstrating the tumor was 0.6 cm away from the active AVF. The excision was performed after AVF disconnection by the vascular surgery team.

### Is a fistula actually a risk factor for SCC?

A total of 118 patients were eligible (25 women and 93 men). Of the total, 80 patients were immunosuppressed (68%) due to kidney, liver, or heart transplantation and 38 were not, and were being followed at the Nephrology department for other kidney diseases. A total of 236 upper limbs were considered, of which 159 had never had a fistula (67%), 43 had an inactive fistula (18.2%) and 34 had an active fistula (14.4%). A total of 164 SCCs were recorded, with an average of 0.69 (minimum zero and maximum 10). Among all SCCs, there was an average of 1.03 in the arms with active fistula; 0.69 with inactive fistula, and 0.62 in those who had never had a fistula.

No statistical significance was found when comparing the occurrence of lesions in the three fistula status groups, (active × inactive p = 0.925; active × never p = 0.0548; inactive × never p = 0.0543).

Considering that having a fistula (active or not) would be the risk factor and that the p-value results from the previous analysis were close to statistical significance, patients with a history of fistula were grouped and compared with those who had never had one, randomizing the choice of one of the arms. Then, statistical significance was found with p = 0.023, demonstrating that having a fistula is a risk factor for the occurrence of SCC.

## Discussion

The occurrence of SCCs very close to AVFs can be a challenge for treatment by dermatologists, requiring a multidisciplinary approach with vascular surgeons, to disconnect the fistula when authorized by the Nephrology team. Considering the cases followed in outpatient clinic, including those reported in this study, a greater frequency of SCCs was observed in limbs that have or have had an active fistula. The literature had already shown that it could be a risk factor, but there is no statistical evaluation in the literature.

The sample showed that a history of AVF is a risk factor for the development of SCC in the affected limb (p = 0.023). Changes in the skin caused by this risk factor, similar to UV exposure, are due to years of dialysis trauma and lymphatic overload, and are not reversible, since even when the fistula no longer functions, the patient still remains at risk of developing SCC in that limb.

This demonstrates the need to pay attention to the formation of tumors in this location, with early treatment of pre-neoplastic and neoplastic lesions, in addition to reinforcing and educating the patient about the need for sunscreen protection.^1^

## Conclusion

Immunosuppressed patients require close monitoring due to the high risk of SCCs. Even greater attention should be paid to patients who have had an AVF, whether active or not, since AVFs are a potential risk factor for SCCs.

## Financial support

None declared.

## Authors’ contributions

Ariany Tomaz de Aquino Saran Denofre: Design and planning of the study; data collection, or analysis and interpretation of data; drafting and editing of the manuscript; collection, analysis and interpretation of data; intellectual participation in the propaedeutic and/or therapeutic conduct of the studied cases; critical review of the literature.

Thais Helena Buffo: Design and planning of the study; critical review of important intellectual content; effective participation in research orientation; intellectual participation in the propaedeutic and/or therapeutic conduct of the studied cases; approval of the final version of the manuscript.

Rafael Fantelli Stellini: Collection, analysis and interpretation of data; effective participation in research orientation; intellectual participation in the propaedeutic and/or therapeutic conduct of the studied cases.

Maria Leticia Cintra: Collection, analysis and interpretation of data; effective participation in research orientation; intellectual participation in the propaedeutic and/or therapeutic conduct of the studied cases.

Renata Ferreira Magalhães: Collection, analysis and interpretation of data; effective participation in research orientation; intellectual participation in the propaedeutic and/or therapeutic conduct of the studied cases.

## Conflicts of interest

None declared.
